# Draft Genomes of Gammaproteobacterial Methanotrophs Isolated from Terrestrial Ecosystems

**DOI:** 10.1128/genomeA.00515-15

**Published:** 2015-06-04

**Authors:** Richard Hamilton, K. Dimitri Kits, Victoria A. Ramonovskaya, Olga N. Rozova, Hiroya Yurimoto, Hiroyuki Iguchi, Valentina N. Khmelenina, Yasuyoshi Sakai, Peter F. Dunfield, Martin G. Klotz, Claudia Knief, Huub J. M. Op den Camp, Mike S. M. Jetten, Françoise Bringel, Stéphane Vuilleumier, Mette M. Svenning, Nicole Shapiro, Tanja Woyke, Yuri A. Trotsenko, Lisa Y. Stein, Marina G. Kalyuzhnaya

**Affiliations:** a San Diego State University, San Diego, California, USA; b Department of Biological Sciences, University of Alberta, Edmonton, Alberta, Canada; c Department of Biology of Extremophilic Microorganisms, Institute of Microbiology and Virology of National Academy of Science, Kyiv, Ukraine; d GK Skryabin Institute of Biochemistry and Physiology of Microorganisms, Russian Academy of Sciences, Pushchino, Russia; e Division of Applied Life Sciences, Graduate School of Agriculture, Kyoto University, Kitashirakawa-Oiwake, Sakyo-ku, Japan; f Department of Biological Sciences, University of Calgary, Calgary, Alberta, Canada; g Department of Biological Sciences, University of North Carolina, Charlotte, North Carolina, USA; h Institute of Crop Science and Resource Conservation–Molecular Biology of the Rhizosphere, University of Bonn, Bonn, Germany; i Department of Microbiology, Faculty of Science, Radboud University, Nijmegen, The Netherlands; j Department of Microbiology, Genomics and the Environment, Université de Strasbourg, CNRS, Strasbourg, France; k Department of Arctic and Marine Biology, UiT The Arctic University of Norway, Tromsø, Norway; l DOE Joint Genome Institute, Walnut Creek, California, USA

## Abstract

Genome sequences of *Methylobacter luteus*, *Methylobacter whittenburyi*, *Methylosarcina fibrata*, *Methylomicrobium agile*, and *Methylovulum miyakonense* were generated. The strains represent aerobic methanotrophs typically isolated from various terrestrial ecosystems.

## GENOME ANNOUNCEMENT

Methane is a potent greenhouse gas (1–3). Methanotrophic bacteria of terrestrial ecosystems contribute to methane sinks not only by mitigating methane emissions but also by consuming atmospheric methane (1–6). Here we report five genomes of gammaproteobacterial methanotrophs isolated from various terrestrial ecosystems. *Methylobacter whittenburyi* (formerly “*Methylobacter capsulatus*” = UCM-B-3033), and *Methylomicrobium agile* (ATCC 35068) are methanotrophic bacteria commonly found in sediment samples from wetlands (7, 8). *Methylobacter luteus* strains (formerly *Methylobacter bovis*, represented here by the strain 98 [IMV-B-3098]) have typically been obtained from meadows, dry hay, and cow mouth samples (7–9). *Methylovulum miyakonense* HT12^T^ (= ATCC BAA-2070) was isolated from a forest soil (10). *Methylosarcina fibrata* AML-C10^T^ (= ATCC 700909) was isolated from a landfill site (11).

The draft genome sequences were generated at the DOE Joint Genome Institute (JGI), using the Illumina (12) and/or PacBio technology (13) (Table 1). Raw reads were assembled using Allpaths, version 39750 (14), Velvet, version 1.1.05 (15) HGAP, version 2.1.1 (16), and/or Phrap, version 4.24 (High Performance Software, LLC). Possible misassemblies were corrected by manual editing in Consed (17–19). All general aspects of library construction and sequencing performed at the JGI can be found at http://www.jgi.doe.gov. Genome annotation was performed using Prodigal (20) and GenePRIMP (21). Additional gene prediction analyses were performed within the IMG (22) and MaGe (23) platforms.

**TABLE 1:** General genome statistics and accession numbers

Species and strain	Sequencing plaform	Genome assembly and annotation	Genome coverage (×)	Genome size (Mb)	No. of scaffolds (no. of contigs)	Core metabolic pathways^*a*^	NCBI accession number
*M. luteus* 98 (= IMV-B-3098)	Illumina, PacBio	Allpaths, Velvet 1/1/05, Phrap 4.24	1,288	5.1	4 (17)	pMMO, Mxa, Xox, FDH, H_4_MTP, H_4_FP, pSC, dPPP, RuMP, EDD, EMP, TCA	ATYJ00000000
*M. fibrate* AML-C10^T^ (= ATCC 700909)	Illumina	Allpaths, Velvet 1/1/05, Phrap 4.24	1,112	5	8 (34)	pMMO, Mxa, Xox, FDH, H_4_MTP, H_4_FP, pSC, dPPP, RuMP, EDD, EMP, TCA	ARCU00000000
*M. miyakonense* HT12^T^ (= ATCC BAA-2070)	Illumina	Allpaths, Velvet 1/1/05, Phrap 4.24	1,199	4.7	9 (32)	pMMO, sMMO, Mxa, Xox, FDH, H_4_MTP, H_4_FP, pSC, dPPP, RuMP, EDD, EMP, TCA	AQZU00000000
*M. agile* ATCC 35068	PacBio	Prodigal, GenePRIMP	210.3	4.5	4 (4)	pMMO, Mxa, Xox, FDH, H_4_MTP, H_4_FP, pSC, dPPP, RuMP, EDD, EMP, TCA	JPOJ00000000
*M. whittenburyi* UCM-B-3033	PacBio	Prodigal, GenePRIMP	209.5	5.4	7 (7)	pMMO, Mxa, Xox, FDH, H_4_MTP, H_4_FP, pSC, dPPP, RuMP, EDD, EMP, TCA	JQNS00000000

a pMMO, membrane-bound methane monooxygenase; Mxa, PQQ-linked methanol dehydrogenases; Xox, PQQ-linked methanol and formaldehyde dehydrogenases; FDH, formate dehydrogenases; H_4_MTP, methanopterin-linked C1 transfer; H_4_FP, folate-linked C1 transfer; pSC, partial serine cycle (i.e., no evidence for glyoxylate regeneration pathway is found); dPPP, dissimilatory pentose phosphate cycle; RuMP, assimilatory ribulose monophosphate pathway; EDD, Entner-Doudoroff pathway, EMP, Embden-Meyerhof-Parnas pathway; TCA, tricarboxylic acid cycle; sMMO, soluble methane monooxygenase.

Genome statistics and predicted core metabolic pathways are shown in Table 1. Genes encoding a soluble methane monooxygenase were detected only in the *M. miyakonense* HT12^T^ genome (24). A functional operon encoding methane monooxygenase was present in all genomes, and a homologous operon encoding related proteins (*pxmABC*) (25) was found in all except *M. miyakonense* HT12^T^. Each genome contains at least one homologue of the large subunit of methanol dehydrogenase (26). Two types of the structural organization of the gene cluster encoding 3-hexulose-6-phosphatesynthase (HPS) and 6-phospho-3-hexuloisomerase (PHI) were found. The genomes of *M. miyakonense* HT12^T^ and *M. fibrata* AML-C10^T^ contain the *hps-phi* operon and another *hpsi* gene encoding an HPS-PHI fused protein (27). *M. luteus* 98 and *M. whittenburyi* UCM-B-3033 possess only the *hps-phi* operon. The genome of *M. agile* ATCC 35068 has only the *hpsi* gene. Genes encoding respiratory nitrate reductase (28) were identified only in the genome of *M. fibrate* AML-C10^T^. The genome sequences indicated that all strains can import and assimilate ammonium (*amtB/glnA/gdhB/ald*) or urea (*urtABCDE*/*ureABCDEFG*) as the sole source of nitrogen. *M. miyakonense* HT12^T^, *M. luteus* 98, and *M. whittenburyi* UCM-B-3033 possess the key genetic elements for nitrogen fixation (*nifKDHWENX*).

Many methanotrophic species (including *Methylobacter* spp.) produce cysts (7). We were not able to identify homologues of known cyst formation genes in any of the sequenced genomes, suggesting that this stage in the life cycle of some methanotrophs might be unique. Production of bacteriocins has been reported for *M. luteus* 98 (29, 30). Two gene clusters encoding a bacteriocin-producing peptidase C39 and a putative precursor (31) were identified in this strain. The contribution of these genes to the production of the biologically active bacteriocin will require experimental validation by mutagenesis studies.

### Nucleotide sequence accession numbers. 

The genome sequences have been deposited in GenBank under the accession numbers listed in Table 1. 
